# The Specificness of Diseases

**Published:** 1856-03

**Authors:** 


*The Specificness of Diseases.* By M. Trousseau. (Clinical Lecture delivered at Hôtel Dieu.) Translated for the Charleston Med. Jour.

In the last century, Natural History could boast of many illustrious names who, by their labors and discoveries, have enlarged the boundaries of knowledge, and rendered their age remarkable. One of the profoundest men of that time, pretending to reduce to a single class all created bein_s, asserted that a reptile, *e. g.*, a frog, finding itself uncomfortable on the hot and sandy soil of Africa, by stretching its neck so as to draw in a fresher and more humid air, would acquire, after many centuries, a considerable dimension of neck, nearly equal to the enormous one of the giraffe. The ribs lengthening, inflecting, and enlarging—the heart of three cavities becoming one of four cavities—and the pulmonary cells becoming completed—a more perfect animal was thus formed. The giraffe was in fact but a perfected frog, which, if it had been put in a marshy pond would have croaked and not brayed.

All this reminds us of what Horace says in his Art of Poetry. It is the fanciful painter placing a woman's head on the shoulders of a horse.

When a physician, so eminent as Broussais, says that the slight catarrhal irritation of the nose occasioned by a very moderate impression of cold, may become an inflammatory coryza, which when aggravated will degenerate into ozœna—when he confounds under the name of enteritis, the slightest diarrhœal flux and the gravest ulceration of the glands of Peyer, I must say, that the theory of progressive development advanced by Lamarck is not more absurd than that which confounds coryza with ozœna, and simple diarrhœa with the pathological lesions of the putrid fever.

All created beings are related. Have not the vertebrata a vertebral column, forming, indistinctly at its superior extremity, an enlargement to contain the encephalon? Does not this vertebral column contain a spinal marrow? Yet, nevertheless, we find some difference between an oviparous animal with a smooth skin and a heart of three compartments, and another with scales and a heart of two compartments. You classify, according to their mode of organization, animals variously placed on the zoological scale; you admit that there are mammifera, birds, reptiles, and fish; then you are forced to admit another class when you are shown that particular species of animals of Oceanica — the opossum and kangaroo — which have a pouch formed of a fold of the skin of the belly to lodge and protect their young. It is the same with the snake tribe. If you are bitten by an adder, you pay little attention to the circumstance; but if struck by a viper, and its venom thrown into the wound, you easily compare the difference between the two bites. To say that the viper is an exaggerated adder, or that the adder is a modified viper, may do in Natural History, but you are forced to make specialization—to make the adder a species distinct from the viper, precisely as you make the vaccine vesicle a peculiar species of the variolous vesicle.

There are in Botany *compteurs de poils*, persons who, always armed with magnifying-glasses and microscopes, find, after studying for a year in one family of plants, a hair or a little gland not before recognized, which discovery they make the subject of a voluminous memoir to be piesented to the Institute. A new name is quickly forged, and the plant recently baptized soon bears the patronym of an officious sponsor. Similar to this is the conduct of the naturalist who does not consider the modifications which heat, light, and other agents exercise over living beings. Place a tad-pole in total darkness and it will remain a tad-pole. The lap-dog and the Newfoundland-dog are both dogs, they perpetuate dogs, but they belong to different varieties of dogs. If we compare the English and Arabian horse, the English and Norman hog, are we not struck with the differences which distinguish them? All these animals have degenerated from a state of nature, but for private and agricultural uses they have improved.

If we enter now the domain of Medicine. and choose our first example in cutaneous Pathology, what we have been trying to explain will be evident. Take an engorgement occurring on the hand: a vesicle appears on the summit; there is pain on pressure; it is the commencement of a furuncle. Several days elapse, the furuncle disappears, but not without having prepared the seed for many others. The furuncle is divided into four parts by the surgeon's knife, the patient suffers accordingly, and there ends the treatment.

In countries where animals have died of pestilential or gangrenous diseases, we may observe the development of a vesicle also, which resembles a furuncle; but on the following day it enlarges, the base becoming œdematous, and the ganglia of the axilla becoming engorged; delirium comes on, and death closes the scene. This furuncle in question—improperly so named—is the *pustule maligne*.

If connection is had with a woman infected with syphilis, inoculation of the virus takes place. Soon there appears a pustule with an enlarged base; then after a time succeeds a roseolous eruption, ulceration of the throat, a demolition of the bones of the nose, etc., etc.

On making an autopsia of a horse which died of glanders, should you prick yourself, you may disregard it; but your eyes will become inflamed; you will be afflicted with a tormenting cough, and an agonizing headache, until delirium supervenes, and you loose consciousness of all exterior things. On opening the dead body, pus is found in the lungs. Glanders can thus destroy by inoculation.

The skin is susceptible to irritation. Cantharides produce a peculiar vesication; mustard causes more pain, but affects the skin in a less degree; nitric acid produces an eschar; the butter of antimony gives rise to terrible suffering. According to each irritant, will be its special irritation. Among workmen who handle phosphorus, the teeth fall out, and the maxillary bones become necrosed. Observe the unhappy potters, glass polishers, and others who work in white and red lead; how pale and stupid they are! how their gums lose their color, and the extensor muscles of their arms become paralyzed! Can you doubt the existence of poisoning by lead?

It was formerly the custom for brewers to put litharge in cider. The Board of Health, charged with the consideration of this important question of the adulteration of beverages, has found litharge in wine and cider. How could they detect the presence of this substance, except by its specific characteristics?

It is scarcely two years since I was called to attend a wood merchant, whom I found affected with lead colic. It was rather uncommon for a dealer in wood to suffer from the effects of lead; so I endeavored to ascertain the cause. On seeing a quantity of shot in his writing desk, I enquired their purpose; and was informed that while writing he amused himself by chewing them as some do tobacco. The Orleans family, not excepting Louis Philippe himself, were once attacked with lead colic at Claremont. M. Gueneau de Mussy discovered that the water used fell from the superior gallery of the château into a reservoir lined with sheet lead.

Each cause produces its specific effects. This is the fundamental law of Therapeutics. Does the intoxication of quinine resemble that of opium? Do the pathological effects of mercury, *e. g.*, salivation and its cutaneous eruptions, resemble those by the poisonous solanaceæ, *e. g.*, stupefaction, dilatation of the pupils, dryness of the throat, dilirium, phantasms, etc? Can you confound the effects of the papaveraceæ, *e. g.*, great confusion of ideas, slight cutaneous eruption, and contraction of the pupils, with those of the solanaceæ? Would you not know how to distinguish a real ulcer from that produced by an epithem of the plant of the family ranunculeæ, the clematis for example? Whilst the bee, by its sting can produce an œdematous fluxion and other disorders with fever, you see other insects produce other effects. Thus, if you touch the nests or the exuviæ which caterpillers leave on trees, your hands and face will become covered with a peculiar eruption. Some persons suffer from cutaneous eruptions after eating mussels, lobsters, and shrimps.

From these illustrations you see, that recognizing the disease, you ascend to the cause; knowing the cause, you descend to the disease. Specific causes have a peculiar character, consistent, invariable, indelible, by which they can be distinguished with certainty.

The specificness is a vital question in Pathology and Therapeutics. In order to convey a clear idea I must give other examples. Nervous diseases are partial or general. With regard to the partial, we have for example a neuralgia of the fifth pair of nerves which returns every day, or every other day, with incredible violence, accompanied by an overflow of tears and coryza; another appearing four or five times daily with painful shootings, photopholia, tears, and coryza; and still another affecting only the inferior maxillary trunk for a minute, with a slight convulsion of the muscles. Of these three neuralgias, the first is very easily cured by the use of quinine, an emetic, a pertubating medication; or even the expectant treatment will also cure this affection which arises from a worm or malarial fever. The second, which is frequently accompanied with chlorosis, is cured by iron; but when it is accompanied by rheumatism, it should be treated with veratine, colchicum, belladonna, etc. The third, the neuralgia with momentary convulsions of the muscles which the seventh pair of nerves supply, is serious, and almost alw ays incurable.

It is very important to know how to distinguish these three kinds of neuralgia. For if confounded the treatment is apt to be unsuccessful. It is dangerous to experiment with remedies, and to administer them at the risk of giving that which is not suitable to the disease. Beware of falling into such an error; for then, losing faith in medicine, you will become a physician of the worst class, a skeptical physician.

Let us now consider general nervous disease. After having waltzed for some time, you have a vertigo; at sea or even on landing you are taken with a vertigo; after being bled vertigo often occurs; a man struck with the *mal d'Hercule* will be attacked with vertigo. If you cannot distinguish these different kinds of vertigo, you may confound epilepsy with the anæmic vertigo, which is consequent upon the loss of blood, and requires iron: vertigo caused by disorder of the stomach will be interpreted by you as a prelude to apoplexy, and you will open a vein; or as a symptom of epilepsy, and you will have recourse to valerian, valerienate of zinc, etc. Whereas if you specialize, when the digestive organs are affected, you will give the carbonate of soda, *eau de vichy*, quassia, etc.; tonics and restoratives to him who is debilitated, and iron to the anæmic. With regard to epilepsy I have remarked that for twenty or thirty minutes it is succeeded by heaviness of head and confusion of ideas. However slight the vertigo of epilepsy may be, it causes confusion in the manifestations of the brain, not only during the attack, but even after it.

Let us now consider the disorders of the mucous membranes. A child is teething; has ganglionic engorgements; its gums are tumefied and ulcerating; and its tongue is swollen. It is suffering, cries continually, refuses to eat and drink, and appears very sick. This state of things, without medication, will cease spontaneously in a week.

In the mouth of another child, near the frænum of the tongue, sometimes on the palatina arches, you perceive a number of small transparent, or greyish vesicles, which after some hours assume a purulent aspect, and which are transformed after the second or third days into painful ulcerations. These are aphthæ, which require but a touch of the nitrate of silver.

You notice, at the insertion of the teeth of a child or an adult, a white streak. You lower the tongue — blood flows along this streak at border — the evil extends, reaching the incisives inferior and superior, and gives to the breath a fœted odor which may last for several months. In families subject to this gingival diphtheritis, the infants generally die of croup.

Gingival diphtheritis predisposes to tracheal diphtheritis. Mercurial stomatitis attacks both sides of the alveoli, and give rise to a periodical periostitis.

Muguet (thrush) in an infant, is a stomatitis characterized by an exudation of small whitish concretions, sometimes disseminated, sometimes confluent, which is always curable in a child of a good constitution. This must not be confounded with muguet of the adult — always a dangerous symptom, which appears in phthisis and cancer, and is a precursor of death. When you see muguet, or simple diphtheritis, how could you give a prognosis unless you specialize? Again; when you see upon the gums of a child of five or six years of age, a small white exudation with redness and tumefaction of the cheek, you know it is an inflammation. The next day the redness is greater, there are several phlyctænæ with black spots in them; the child will die with gangrene of the mouth. You should learn to distinguish the affection of the buccal mucous membrane, which are as distinct from each other as variola is from measles, as measles is from scarlatina, as scarlatina is from ecthyma, etc. The child with gangrene of the mouth, if the tooth be extracted and the gums be well cauterized, will recover; but if this be not promptly done it will surely die. Muguet is to be treated with borax; mercurial stomatitis with the chlorate of potash.

Apropos to the disease of the throat, where so much confusion exists, you will see diphtheritic inflammations, which remain stationary; diphtheritic inflammation of the tonsils not extending farther; and herpetic diphtheritis which, after having assailed the tonsils, extend into the larynx. But there are cases where you will see an exudation of a light yellow color occupying a portion of a tonsil, then the entire tonsil, then the uvula, and the posterior wall of the pharynx. There is cough, which becomes hoarse, croupy, and is finally extinct. You perform tracheotomy, and you find false membrane.—There is then an angina which is circumscribed and stationary, and others which have a tendency to spread over the neighboring parts. You should clearly distinguish the angina which can be cured by borax, alum, or the bicarbonate of soda, from diphtheritic angina. Do not resort to useless and dangerous remedies, for the relief of the former; nor boast of success, when you have relieved, by heroic means, an angina which would have got well without you.

It is not possible to practice medicine successfully without a specialization of diseases; but I do not contend, as some have erroneusly supposed, that a specific disease must necessarily require a specific remedy. I know very well that the four kinds of ophthalmia can be cured by the same means; that two diseases, essentially different, as the ordinary furuncle and the *pustule maligne*, can be arrested by the actual cautery; and that the variolous pustule and that of ecthyma can be destroyed by cauterization.

However it may be regarded, the specificness of diseases is of incalculable value to the practitioner; without it he must follow the beaten track of the blindest empiricism.—*Gazette des Hôspitaux*, *July*, 1854.

